# Regulation of transforming growth factor-β signalling by SUMOylation and its role in fibrosis

**DOI:** 10.1098/rsob.210043

**Published:** 2021-11-10

**Authors:** Xinyi Wang, Ting Liu, Yifei Huang, Yifeng Dai, Hui Lin

**Affiliations:** ^1^ First Clinical Medical School, Nanchang University, Nanchang 330006, Jiangxi Province, People's Republic of China; ^2^ Department of Pathophysiology, School of Basic Medical Sciences, Nanchang University, Nanchang 330006, Jiangxi Province, People's Republic of China; ^3^ Second Clinical Medical School, Nanchang University, Nanchang 330006, Jiangxi Province, People's Republic of China

**Keywords:** transforming growth factor-β, SUMOylation, fibrosis

## Abstract

Fibrosis is an abnormal healing process that only repairs the structure of an organ after injury and does not address damaged functions. The pathogenesis of fibrosis is multifactorial and highly complex; numerous signalling pathways are involved in this process, with the transforming growth factor-β (TGF-β) signalling pathway playing a central role. TGF-β regulates the generation of myofibroblasts and the epithelial–mesenchymal transition by regulating transcription and translation of downstream genes and precisely regulating fibrogenesis. The TGF-β signalling pathway can be modulated by various post-translational modifications, of which SUMOylation has been shown to play a key role. In this review, we focus on the function of SUMOylation in canonical and non-canonical TGF-β signalling and its role in fibrosis, providing promising therapeutic strategies for fibrosis.

## Graphical abstract

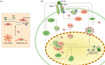

## 1. Introduction

Transforming growth factor-β (TGF-β) is a pleiotropic cytokine that regulates a wide range of biological processes, such as proliferation, differentiation, migration and metabolism [1]. In the canonical pathway, the TGF-β ligand first binds and activates the TGF-β type II receptor (TβRII), which in turn recruits and phosphorylates TGF-β type I receptor (TβRI) [1]. TβRI phosphorylates the C-terminal serine residue of receptor-activated Smads (R-Smads, including Smad2 and Smad3), which form a heteromeric complex with Smad4 (Co-Smad) to enter the nucleus [2]. The complex then associates with other transcription factors to positively or negatively regulate the transcription of target genes [3]. Inhibitory Smads (I-Smads, including Smad6 and 7) block interaction between R-Smads and TβRI by competing with R-Smads following association with activated TβRI [4]. In addition, I-Smads act as inhibitors by recruiting the E3 ubiquitin ligase Smad ubiquitination regulatory factor 2 (Smurf2) to degrade activated TβRI via ubiquitination [5]. The various functions of TGF-β rely on the transcription of downstream genes and cross-talk with other signalling pathways [6]. It is worth noting that post-translational modification (PTM) is involved in TGF-β pathway regulation [7], and the role of SUMOylation in the TGF-β signalling pathway is attracting increasing attention [8]. SUMOylation modulates signal transduction by altering the subcellular localization, protein–DNA binding and ubiquitin-dependent degradation of target substrates [9].

Four small ubiquitin-like modifiers (SUMOs) have been identified: SUMO1, SUMO2, SUMO3 and SUMO4 [10]. SUMO1 is mainly present under physiological conditions, whereas SUMO4 is present under pathological conditions. Levels of SUMO2 and SUMO3 are elevated by stress [11]. In addition, SUMO5 has been identified and shown to be involved in the formation and destruction of promyelocytic leukaemia nuclear bodies (PML-NBs) [12]. SUMOs are activated by E1 ubiquitin-activating enzymes composed of activation of Smt3p 1 (Aos1) and ubiquitin-activating enzyme 2 (Uba2) in an ATP-dependent manner and then translocate to E2 Ubc9 and interact with target protein residues with the assistance of Ubc9. Binding usually occurs at a lysine within the consensus sequence ΨKx (D/E), where Ψ represents a large hydrophobic residue, but the modification may occur on other individual lysines if there is no consensus sequence [8,13]. The SUMO-E3 ligase recognizes substrates and promotes transfer of the SUMO protein from Ubc9 to the target protein [14]. Interestingly, the SUMO-conjugating enzyme E2 can also be SUMOylated at sites Lys-48 and Lys-49; the binding of SUMO protein to Lys-49 promotes interaction of Ubc9 with SUMO interaction motif (SIM)-containing proteins, which can be further enhanced when the SIM-containing proteins are SUMOylated [15]. SUMOylation is a completely reversible enzymatic reaction, and SUMO proteins can be removed from target proteins by SUMO-specific proteases (SENPs) [16]. Seven SENPs (SENP1–3 and SENP5–8) have been identified [17], and SENP family members can not only reverse the modification but also cause maturation of pro-SUMO to conjugatable SUMO via a modification [18].

## 2. Transforming growth factor-β regulation in fibrogenesis

Fibrosis refers to the pathology in which the structure of a damaged organ is repaired, but the function is not restored. Fibrosis is attributed to excessive deposition of extracellular matrix (ECM) caused by chronic inflammation, which is stimulated by infection, autoimmune reactions and physical or chemical stimulation [19]. Myofibroblast transdifferentiation and matrix accumulation are the two major pathophysiological mechanisms driving fibrosis [20]. In healthy organs, the composition and dynamic structure of the ECM rely on matrix metalloproteinases (MMPs) [21]. Views about the role of TGF-β in regulating ECM and MMP expression differ. Some studies have shown that the TGF-β/Smad receptor 1 inhibitor GW788388 increases MMP9 and improves cardiac fibrosis [22], but others have indicated that TGF-β1 can stimulate MMP2 and MMP9 activity. TGF-β has been shown to stimulate MMP activity, but this effect only occurs in rats and mice and not in fibrotic human organs [23]. In addition, TGF-β1 can promote myofibroblast (α-SMA is a marker of mature myofibroblasts) differentiation in fibroblasts, endothelial cells and epithelial cells [24–26], ultimately leading to ECM deposition. As mentioned above, TGF-β promotes fibrogenesis by regulating fibrotic gene expression and fibroblast differentiation [27].

TGF-β inhibitors include anti-TGF-β1 neutralizing antibodies that prevent the binding of ligands and receptors, anti-TGF-β receptor antibodies, inhibitors that block transcription and translation of TGF-β and inhibitors that prevent phosphorylation of mediators downstream of TGF-β, such as Smad3 and Smad4 [28]. Although multiple studies have indicated the therapeutic efficacy of these inhibitors in fibrotic mice [29], few clinical effects on fibrosis have been reported through TGF-β pathway targeting. Some studies attribute this effect to wide TGF-β expression in normal cells; others suggest that simply inhibiting interaction between ligands and receptors prevents activation of Smad7, leading to imbalance in profibrotic negative self-regulation [28,30]. Moreover, inhibiting SUMOylation prevents systemic sclerosis (SSc) in preclinical models [31]. Therefore, SUMOylation-mediated regulation of TGF-β signalling may provide new antifibrotic strategies.

## 3. The role of SUMOylation in the canonical transforming growth factor-β pathway

TβRI is the only receptor of TGF-β signalling that has been demonstrated to be SUMOylated. TβRI is SUMOylated at lysines 385 and 389 (Lys-385 and Lys-389), with the latter being the major site [8]. SENP2 reverses this modification, and SENP2 overexpression suppresses the TGF-β-induced epithelial–mesenchymal transition (EMT) [32]. This effect may be attributed to changes in the structure of TβRI [33]. Phosphorylation of R-Smad requires the L3 loop and adjacent α-helix1 in the C-terminal MH2 domain to bind to the L45 loop and glycine and serine (GS) region of TβRIs. Lys-389 is located at the surface of the kinase domain and has the same orientation as the GS region [8]. Therefore, SUMOylation of TβRI is likely to affect Smad3 activation, and this modification enhances interaction between Smad3 and TβRI and promotes Smad3 C-terminal phosphorylation [34]. Under TGF-β stimulation, fibroblasts expressing K389R TβRI show reduced transcription of a Smad3-responsive promoter and reduced Smad7 mRNA expression compared with cells expressing wild-type TβRI. This finding indicates that SUMOylation of TβRI contributes to the response of cells to TGF-β [34]. The study also demonstrated that SUMOylated TβRI blocks the fibroblast proliferations, which may be attributed to enhanced TGF-β signalling inhibiting fibroblast growth factor (FGF)-mediated regulation of fibroblast division and proliferation. Nevertheless, by preventing myofibroblast differentiation, FGF is recognized as protecting against lung fibrosis [35,36]. These studies indicate that SUMOylation regulates fibrogenesis mainly by altering the transcription of EMT- and ECM-related genes rather than by promoting fibroblast proliferation.

**Figure 1.**
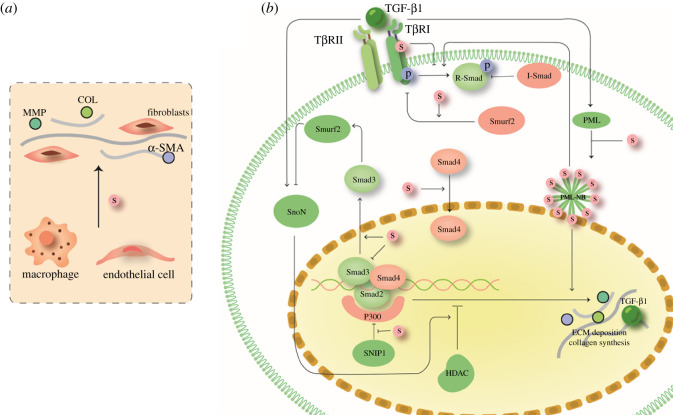
The role of SUMOylation in the canonical TGF-β signalling pathway. (*a*) The TGF-β signalling pathway plays an important role in tissue fibrosis. SUMOylation modifies the type I receptor, inhibits phosphorylation of R-Smad and promotes the inhibitory effect of Smurf2 on this process. SUMOylation also promotes nuclear export of Smad3 and nuclear import of Smad4. In addition, Smad nuclear interacting protein 1 (SNIP1) is modified by SUMO to inhibit the production of fibrosis-related proteins; SUMOylated PML promotes the production of related proteins. COL, collagen. (*b*) SUMOylation participates in myofibroblast (α-SMA is a marker of mature myofibroblasts) differentiation from endothelial cells and macrophages. HDAC, histone deacetylase.

Phosphorylated Smad3 binding to the AP-1 site is indispensable for responses of both MMP-1 and TIMP-1 to TGF-β, inhibiting expression of MMP and further inducing ECM deposition [37]. By stimulating the nuclear export of Smad3, SUMOylation reduces the binding of Smad3 to DNA [38]. SUMOylation of the MH2 domain of Smad3, which is mediated by PIASy, has been demonstrated to prevent TGF-β-induced Smad3 phosphorylation [39]. After forming a complex with Smad4, an important mediator that shuttles between the nucleus and the cytoplasm and has also been shown to be SUMOylated, Smad3 enters the nucleus and regulates genes involved in fibrosis [40]. In Smad4, Lys-113 in the MH1 domain and Lys-159 in the linker segment serve as SUMOylation sites [41]. Nonetheless, the role of Smad4 SUMOylation in the regulation of TGF-β transcription remains controversial with different conclusions on the role of SUMOylation in controlling Smad4 activity and stability [42,43]. Some researchers support a negative role of Smad4 SUMOylation in TGF-β signalling because the K113R/K159R mutation reduces the polyubiquitination of Smad4 [43], though others support a positive role for Smad4 SUMOylation due to increased Smad4 activity [44,45]. These opposing conclusions may be caused by different cell contexts (figure 1).

## 4. The role of SUMOylation in the non-canonical transforming growth factor-β pathway

**Figure 2.**
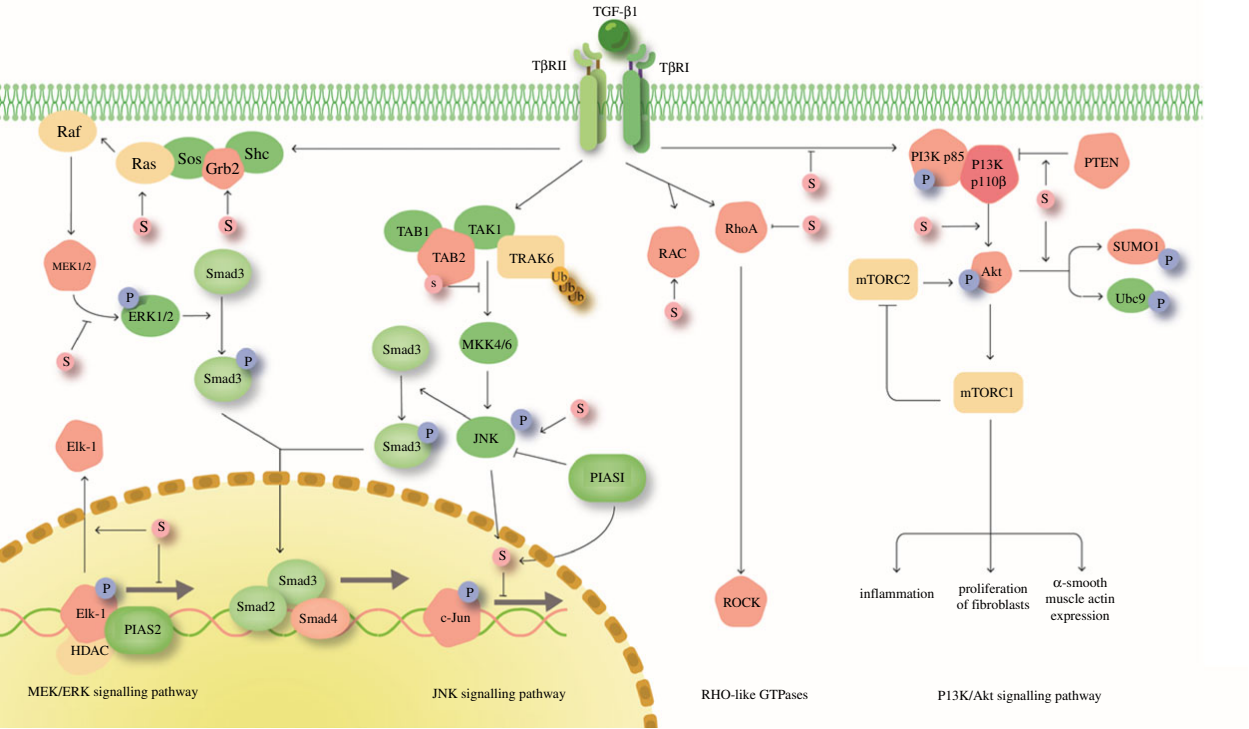
The role of SUMOylation in the non-canonical TGF-β signalling pathway. MEK/ERK signalling: SUMOylation inhibits activation of TAB2 and MEK and promotes Elk-1 export to prevent its transcription. JNK signalling: SUMOylation prevents TAB2 complex activation of MKK4/6 while promoting JNK phosphorylation. Transcription of the downstream JNK gene *c-JUN* is blocked by SUMOylation. Rho-like GTPases: RAC and RhoA activity is promoted by SUMOylation. PI3K/Akt signalling: SUMOylation directly inhibits PI3K phosphorylation and enhances negative regulation of PI3K by PTEN. SUMOylation also induces Akt to promote SUMO1 and Ubc9 activation.

In addition to the Smad signalling pathway, SUMOylation plays a crucial role in non-Smad signalling-mediated fibrogenesis (figure 2). Non-Smad signalling pathway components include mitogen-activated protein (MAP) kinases (ERK, p38 and JNK), phosphatidylinositol-3-kinase (PI3K) and Rho-like GTPases [46].

## 5. MEK/ERK

Activated TβRI receptors have been reported to phosphorylate tyrosine and serine residues in the ShcA protein and to induce the association of ShcA with Grb2 and Sos [47]. Guanosine diphosphate-bound RAS switches to activated guanosine triphosphate (GTP)-bound RAS under Sos stimulation, and activated RAS further phosphorylates MEK1 and ERK [48]. ERK is involved in regulating TGF-β receptor expression and ECM production. Inhibiting ERK phosphorylation is beneficial for reducing levels of α-SMA [26], collagen-1 (COL-1) and fibronectin in scar tissues [49]. Furthermore, inhibiting ERK activation improves fibrosis by suppressing the transition of fibroblasts to myofibroblasts induced by TGF-β1 [50,51]. This finding confirms that the RAS–RAF–MEK–RK signalling cascade promotes fibrogenesis [52]. Grb2 and MEK, important members of the TGF-β signalling pathway, are also modified by SUMO. Conjugation of SUMO to Lys-56 in Grb2 promotes the motility and transformation of the fibroblast cell line NIH/3T3, and this modification induces Grb2–Sos1 complex formation, contributing to activation of the MEK/ERK pathway [53]. The function of Ras SUMOylation is similar to that of Grb2, and both facilitate activation of MEK and ERK in downstream signals [54]. However, SUMOylated Ras has mostly been investigated with regard to anti-cancer activity, and no studies have reported its role in fibrosis [55]. MEK1 and MEK2 SUMOylation occurs at Lys-104 and Lys-108, which disturbs binding between MEK and ERK, further inhibiting phosphorylation of the latter [56,57].

Elk-1 is a crucial downstream factor for MEK–ERK; the loss of Elk-1 would elevate the level of type I and type III collagens and lead to fibrosis of internal organs owing to the loss of repressed integrin expression [58,59]. PIAS protein acts as a coactivation protein to derepress transcription through facilitating the loss of the repressive histone deacetylase (HDAC-2) from SUMOylated Elk-1 [60]. SENP1 is involved in Elk-1 deSUMOylation process [61]. Elk-1 SUMOylation at Lys-230, Lys-249 and Lys-254 promotes its shuttling from the nucleus to the cytoplasm [62]. SUMOylation of Elk-1 recruits HDAC-2 to promoters, which prevents targeted transcription [63], and ERK activation leads to the loss of Elk-1 SUMOylation, which promotes Elk-1 activation [64]. Overall, the modification of Grb2 with SUMO enhances MEK/ERK pathway component transcription while inhibiting MEK and Elk-1 activity. Furthermore, ERK–mitogen-activated protein kinase (MAPK) independently participates in Smad3 phosphorylation [65]. Inhibiting the MEK/ERK pathway attenuates the stimulatory effect of TGF-β1 on Smad3 but has a slight effect on Smad4 activity [66]. ERK promotes Smad3 phosphorylation, possibly via ERK activation [67]. One study showed that Smad3 acts as a negative regulator inhibiting TGF-β-induced EMT [68]. Regardless, it remains to be determined whether SUMO modification inhibits the phosphorylation of Smad3 by ERK and leads to organ fibrosis.

## 6. PI3K/Akt

In normal fibroblasts, phosphatase and tension homologue (PTEN) is involved in inhibiting PI3K/Akt/mTOR activation. Conversely, inappropriately low PTEN activity enhances interaction between fibroblasts and polymerized collagen, which leads to pathological activation of PI3K/Akt in idiopathic pulmonary fibrosis [69]. Studies have shown that the abnormal activation of the PI3K/Akt signalling pathway is closely associated with the occurrence of fibrosis. Moreover, PI3K/Akt is associated with endoplasmic reticulum stress to induce fibrogenesis, indicating that treatment with PI3K inhibitors may reduce fibroblast proliferation and improve fibrotic organ function [70]. In addition, mTOR activation by Akt may participate in fibrogenesis by enhancing chemotaxis of alveolar macrophages and proliferation of fibroblasts [71,72]. These cells are recruited to damaged tissue and release TGF-β1, which ultimately leads to fibrosis [73]. Consequently, inhibiting PI3K/Akt/mTOR may be a strategy for ameliorating fibrosis [74].

PI3K and Akt have been demonstrated to serve as substrates modified by SUMO proteins. PI3K is composed of a p110 catalytic subunit and p85 regulatory subunit, and TGF-β cell surface receptors activate p85 to catalyse the conversion of phosphatidylinositol 4,5-bisphosphate (PIP2) to phosphatidylinositol 3,4,5-triphosphate (PIP3) [75]. SUMOylation of p85 inhibits its activation and prevents cell migration and transformation [76]. SUMOylation of p110β stabilizes the protein and increases its activation of Akt [77]. Akt activation is promoted by SUMO-E3 ligase PIAS1 and reversed by SENP1, SENP2 and SENP3 [78–80]. Akt is SUMOylated at Lys-276 and Lys-301, which enhances its regulatory function at the G1/S transition during cell cycle progression, cyclin D1 expression and cell proliferation [81,82]. Fibronectin is an adhesion molecule that plays an important role in wound healing and is involved in ECM remodelling in fibrosis, and increasing Akt SUMOylation levels enhances its capacity to regulate fibronectin splicing patterns [83]. Interestingly, Akt SUMOylation promotes phosphorylation of Ubc9 and SUMO1 and regulates global SUMOylation [82]. PTEN acts as an inhibitor of PI3K/Akt through dephosphorylation of PIP3 [84]. SUMO1 binds to Lys-254 and Lys-266 sites in the C2 domain of PTEN, which promotes its nuclear localization [85,86]. PIASxα increases PTEN protein stability by reducing PTEN ubiquitination, which leads to G0/G1 cell cycle arrest and suppresses cell proliferation [87]. In summary, SUMOylation weakens PI3K and enhances PTEN to promote and inhibit PI3K/Akt pathway activity, respectively. In general, SUMOylation of PI3K/Akt signalling pathway components is involved in regulating fibrosis progression, but the specific mechanism remains to be elucidated.

## 7. JNK

JNK activation mediates fibrosis, which correlates with TGF-β-induced EMT and activated fibroblast production of collagen [88]. TGF-β binds to tumour necrosis factor receptor (TNFR)-associated factor 6 (TRAF6) to induce K63-linked ubiquitination of TRAF6, facilitating recruitment of the specific binding partners TAK1-binding proteins (TAB1/2/3) to activate TAK1 [89]. Activated TAK1 phosphorylates MKK [90], and MKK4/7 are potential activators of JNK [91]. JNK phosphorylation plays a crucial role in α-SMA and Col1A1 production, suggesting that regulating JNK activation is a strategy to attenuate fibrosis [92]. TAB2 has been found to be modified by SUMO1 at Lys-329 and, with the assistance of PIAS3, inhibits activation of TAK1 [93]. One study has demonstrated that TAB2 modification by SUMO1 at Lys-329 with the assistance of PIAS3 inhibits the activation of TAK1 [93]. However, this modification only reduces TAB2 activity and does not affect its subcellular localization [93]. The level of p-JNK is decreased by SENP1, whereas SUMO-1 overexpression increases phosphorylation of JNK, which indicates that SUMOylation also participates in regulating JNK activation under oxidative stress conditions [94]. Overall, SUMOylation has a positive effect on the phosphorylation of JNK.

Rather than acting as a positive regulator of the JNK signalling pathway in human embryonic stem cells, PIAS1 antagonizes JNK activity independently of its ligase function [95]. Furthermore, PIAS1 is phosphorylated in response to JNK activation, which disturbs the SUMOylation–deSUMOylation balance. For example, PIAS1 enhances SUMOylation of *c-Jun*, a major downstream target in the JNK pathway, in a ligase-independent manner [95]. The transcription factor *c-Jun* is part of the activator protein 1 (AP-1) complex, and attenuating *c-Jun* activity reduces the expression of AP-1-dependent inflammatory genes in both monocytes and epithelial cells [96]. Studies have shown that the loss of phosphorylation sites in *c-Jun* aids its binding with SUMO1. Additionally, a SUMO1-deficient *c-Jun* K229 mutant exhibits higher transactivation potential at AP-1-containing promoters than wild-type *c-Jun*, which indicates that SUMO1 serves as a negative regulator of *c-Jun* activity [97]. These results can be partially explained by competition between SUMOylation and phosphorylation [97]. In summary, SUMOylation attenuates TAB2 and *c-Jun* activation but promotes JNK activation.

## 8. Rho

RhoA is a Rho GTPase that belongs to the family of Ras-related small GTP-binding proteins [98]. Only GTP-bound Rho is able to activate downstream Rho-associated coiled-coil-containing kinase, leading to ECM deposition and fibrosis following phosphorylation of myosin phosphatase [99]. In addition, activated RhoA signalling mediates scleral myofibroblast differentiation and hepatic stellate cell (HSC) proliferation, migration and activation [100,101]. HEK293T cells co-transformed with SUMO2/3 developed cell protrusions and pseudopodia, suggesting that the activity of RhoA may be inhibited by SUMO modification, as increased RhoA activity inhibits axon regeneration [102,103]. Nevertheless, the role of RhoA SUMOylation in fibrosis remains unclear. Rac is another member of the Rho GTPase family and has previously been shown to antagonize RhoA activity in mouse embryonic fibroblasts (MEFs) [104]. Ablation of the *Rac1* gene increases the expression of α-SMA [105], which suggests that Rac is a novel therapeutic target against progressive fibrosis [106]. As Rac1-null MEFs derived from Rac1 conditional knockout mice are defective in cell migration, Rac may conjugate to SUMO1 with the help of PIAS3 to promote cell migration and invasion [107]. SUMOylation is beneficial for the stabilization of a pool of GTP-bound Rac1; thus, SUMOylation promotes Rac activation, and SENP deSUMOylates Rac1 [107]. Overall, the effects of SUMO modification on the RhoA signalling pathway need further investigation.

## 9. Others

Other transcription-related factors participate in the regulation of TGF-β signal transduction through SUMO modification.

## 10. Promyelocytic leukaemia protein

PML protein was originally identified as a fusion partner of retinoic acid receptor alpha in patients with acute promyelocytic leukaemia, but it has become an emerging factor in cancer owing to its role in the regulation of apoptosis, protein modification and cellular senescence [108]. Studies have shown that PML is also involved in fibrosis regulation. The B-box domain of cytoplasmic PML interacts with the MH1 domain of Smad2/3 to promote Smad2/3 phosphorylation and the production of TGF-β1 [109], and the overexpression of TGF-β1 increases activation of myofibroblasts and the deposition of ECM [110]. TGF-β1 further promotes generation of PML to form a positive feedback loop. It is worth noting that SUMOylation also participates in the abovementioned regulation. PML SUMOylation is promoted by SUMO-E3 ligase PIAS1 and RanBP2 and reversed by SENP2/5/6 [111,112]. PML is conjugated to SUMO1/2/3 on Lys-65, Lys-165 or Lys-490 [15], and Lys-65 mutation affects SUMOylation at other sites and leads to a low level of PML. Consequently, Lys-65 is considered to be the key site for SUMOylation [113]. SUMOylation at Lys-65 and Lys-160 leads to degradation of PML; SUMOylation at Lys-490 contributes to the formation of stable PML-NBs [111]. Indeed, PML SUMOylation is necessary for PML-NB formation and recruitment of Daxx and ring finger protein 4 (RNF4) to PML-NBs [114]. RNF4, a SUMO-targeted ubiquitin E3 ligase, targets SUMO-modified PML for ubiquitin-mediated degradation [115]. Studies have shown that silencing RNF4 induces liver fibrosis through the accumulation of SUMOylated PML. Furthermore, silencing Ubc9 suppresses protein and mRNA expression of TGF-β1 to inhibit the TGF-β/Smad pathway as well as the expression of phosphorylated Smad2/3 and α-SMA. These results suggest that PML SUMOylation triggers HSC activation by increasing TGF-β signalling, thereby promoting the production of collagen I and α-SMA [116].

## 11. SnoN

SnoN (Ski novel protein), a member of the Ski family of proteins, was initially identified as a nuclear proto-oncoprotein based largely on its close homology to v-ski, the transforming protein of avian Sloan–Kettering retrovirus [117]. The complex roles of Ski and SnoN in tumorigenesis and embryonic development have been researched extensively [118,119]. SnoN, a novel negative regulator of TGF-β/Smad signalling, is depleted by Smurf2-mediated polyubiquitination and degradation within the context of fibrosis, ultimately contributing to inhibition of myofibroblast function and phenotypic conversion [120]. It is worth noting that Smad3 downregulates the expression of SnoN by elevating Smurf2 protein levels, indicating interaction between SnoN and TGF-β [121]. SnoN is also regulated by PTM. TAK1 mediates phosphorylation of SnoN and promotes ubiquitination and degradation of SnoN [122]. However, SUMOylation of SnoN at Lys-50 and Lys-383 with the help of PIASs is unrelated to its ubiquitination and does not alter its stability or subcellular localization. SnoN is regulated by SUMOylation, leading to the repression of TGF-β signalling-mediated transcriptional activity in a promoter-specific manner [123]. SnoN degradation via ubiquitination is mediated by Smurf2 [124], an E3 ubiquitin ligase shown to be SUMOylated at Lys-26 and Lys-369, which modulates its stability and induces TβRI degradation to prevent TGF-β-induced EMT [125]. Therefore, SUMO modification may have an indirect regulatory effect on SnoN.

## 12. Smad nuclear interacting protein

Smad nuclear interacting protein 1 (SNIP1) is an evolutionarily conserved protein composed of 396-amino acid nuclear proteins that contains a bipartite nuclear localization signal and a Forkhead-associated domain [126]. Smad1/2 interacts with the carboxyl terminus of SNIP1, whereas Smad4 interacts with the amino terminus of SNIP1; the interaction of SNIP1 and Smad4 is stronger and more direct [127]. Studies have demonstrated that SNIP1 prevents ligand-dependent transcription by restricting the interaction between the Smad2/3–Smad4 complex and CBP/p300. However, SUMO modification of Lys-5, Lys-30 and Lys-108 of SNIP1 antagonizes its inhibitory effect on TGF-β signalling. Lys-30 is regarded as the major SNIP SUMOylation site, and SUMO modification of SNIP1 is enhanced by the SUMO-E3 ligase PIAS protein and inhibited by the SUMO protease SENP1/2. TGF-β treatment results in reduced production of MMP2 in SNIP1 (K5R/K30R/K108R) mutant-expressing cells compared with wild-type SNIP1 [128]. Smad-mediated MMP2 serves as a key ligase for preventing overproduction of the ECM, and elevated expression of MMP2 improves fibrosis [129]. Therefore, the ability of SNIP1 to block formation of the Smad complex and prevent interaction between p300 and Smads is impaired by SUMOylation, which ultimately enhances TGF-β-induced cell migration and invasion [128].

## 13. DeSUMOylation as a novel strategy for fibrosis treatment

Considering the significance of protein SUMOylation in TGF-β signalling regulation (table 1), the SUMOylation pathway is a promising therapeutic target for clinical fibrosis drug discovery. Numerous compounds have been designed as SUMO inhibitors. These compounds can be divided into three categories according to their mechanism of action. First, compounds are SENP inhibitors that inhibit maturation and deSUMOylation [130]. Second, compounds are SUMO mimics represented by multivalent poly-SUMO chain inhibitors [131]. Third, compounds are inhibitors of SUMO enzymes. Small-molecule inhibitors targeting SUMO-E1/SUMO-E2 enzymes have been found as natural products or designed through chemical synthesis. However, to date, no small-molecule inhibitor has been designed to effectively inhibit SUMO-E3 enzymes [132]. Ginkgolic acid (GA) is the most widely used and commercially available chemical, which inhibits the SUMOylation modification pathway by blocking formation of the SAE-SUMO intermediate [133]. SUMOylation of Smad4 and PML is repressed by GA [134]. Although studies on the application of GA have mostly focused on tumours, the use of GA to alleviate myocardial infarction-induced cardiac fibrosis is promising [135]. GA suppresses the expression of EMT-related genes through inhibition of SUMO conjugation to inhibit fibrosis [135,136]. This may be explained by the vital role of EMT in the occurrence and progression of cancer and fibrosis. Nonetheless, adverse reactions such as allergic reactions limit the GA content in drugs [137]. GA also inhibits other biological processes, which complicates its use as an inhibitor of SUMOylation [138]. Some scholars propose that ‘extending new clinical applications to old drugs, which have been confirmed as inhibitors targeting SUMO pathway, would be a solution presenting few novel side effects' [132]. However, the dose dependence and low selectivity of these drugs is a problem [131]. Thus, finding structure–activity relationships in designing high-selectivity SUMO inhibitors is very important. The effects of SUMOylation on different substrate proteins vary according to the role of modification in canonical and non-canonical TGF-β signalling, which might contribute to the structure of the substrate proteins and their locations in the pathway. Assessment of which molecular mechanisms determine SUMOylation resistance or sensitivity for a substrate should be considered in the search for combination therapies, which would reduce adverse reactions and the possibility that SUMO inhibitors bind to other molecules in addition to targets [131]. Most related drugs found now are global SUMO inhibitors. Using these drugs promotes cell injury after ischaemia, which leads to fibrogenesis [139], suggesting that further exploration of specific SUMO isoforms in different diseases will facilitate the development of highly selective SUMO inhibitors and improve clinical antifibrosis therapy [140]. Specific isoforms associated with the abovementioned proteins are summarized in table 2.

**Table 1.** SUMOylated substances in TGF-β signalling pathway.

protein	SUMOylation site	SUMOylation	E3 enzyme	deSUMOylation enzyme	effects on biological responses	reference
TβRI	Lys-385, Lys-389	SUMO1	unclear	SENP2	enhance recruitment and phosphorylation of Smad3	[8]
Smad3	MH2	SUMO1	PIASy	unclear	inhibits activation and nuclear export of Smad3	[38]
Smad4	Lys-113, Lys-159	SUMO1/2/3	PIAS1, PIASxα, PIASxβ, PIASy	SENP1, SENP2	enhance nuclear recruitment of Smad4; inhibits or promotes activity and stability of Smad4	[43]
Grb2	Lys-56	SUMO1	unclear	unclear	promotes binding of Grb2 and Sos1; induces activation of downstream signalling	[53]
Ras	Lys-42	SUMO3	PIASy	SENP1, SENP2	promotes activation of ERK	[54]
MEK1/MEK2	Lys-104/Lys-108	SUMO1	MEKK1 (MEK1 unique E3 enzyme)	unclear	prevent binding of MEK and ERK to inhibit activation of ERK	[56]
Elk1	Lys-230, Lys-249, Lys-254	SUMO1/2/3	PIASxα (in an E3 activity-independent manner	SENP1	regulate nuclear shuttling; simulates HDAC and PIAS2 to inhibit ELK-mediated transcription	[56,60–62]
PI3K	P85 (including Lys-535, Lys-592), P110(Lys-952)	SUMO1/2	unclear	unclear	inhibit phosphorylation of PI3K and its activation and downstream signalling	[76]
Akt	Lys-276, Lys-301	SUMO1	PIAS1	SENP1, SENP2, SENP3	enhance activity of AKT; enhances PTEN SUMOylation; induces phosphorylation of SUMO1 and Ubc9	[78–80,82]
PTEN	Lys-254, Lys-266	SUMO1	PIASxα	SENP1	promote nuclear localization of PTEN; inhibit the PTEN/PI3K/AKT pathway.	[85–87]
Tab2	Lys-329	SUMO1	PIAS3	unclear	inhibits activity of Tab2	[93]
JNK	unclear	unclear	PIAS1	unclear	inhibits phosphorylation of JNK and prevents its activity	[95]
RhoA	unclear	SUMO2/3	unclear	unclear	inhibits activity of RhoA	[103]
RAC	unclear	SUMO1	PIAS3	unclear	promotes activation of RAC, leading to defects in embryonic fibroblast migration	[107]
PML	Lys-65, Lys-165, Lys-490	SUMO1, SUMO2, SUMO3	PIAS1, RanBP2	SENP2/5/6	promote formation of PML-NBs; enhance P-Smad2/3 and TGF-β1 mRNA expression	[15,111,112]
SnoN	Lys-50, Lys-383	SUMO1	PIAS1, PIASy	unclear	inhibit TGF-β-induced EMT but do not change ubiquitination degradation, stability or subcellular localization	[122,123]
SNIP1	Lys-5, Lys-30, Lys-108	SUMO1	PIAS1, PIAS3, PIASxα and PIASxβ	SENP1/2	inhibit the negative effect of SNIP1 on MMP2 to enhance TGF-β transcription	[128]

**Table 2.** Specific SUMO isoforms linked to components of the TGF-β signalling pathway.

SUMO isoforms	type of effects	substrate proteins	effect	references
SUMO1	positive	TβRI	Smad3 activation	[8]
		Smad4	transcriptional response superfamily	[41,42,44]
			nuclear accumulation	
			stability	
		Grb2	Ras/MEK/MAPK pathway activation	[53]
		PTEN	promote nuclear localization	[85,86]
			binding to the plasma membrane	
			inhibition of the PTEN/PI3K/AKT pathway	
		Akt	activation	[79,82]
			enhances PTEN SUMOylation	
		JNK	JNK activation	[94]
		RAC	promotes activation of RAC	[107]
		SnoN	inhibits EMT	[123]
	negative	Smad3	DNA-binding activity	[38]
		Smad4	transcriptional activity	[43]
		MEK1/2	ERK activation	[57]
		TAB2	inhibits activity of TAB2	[93]
		RhoA	inhibits activity of RhoA	[103]
		SNIP1	inhibits inhibitory activity of SNIP1	[128]
SUMO2/3	positive	Smad4	TGF-β signalling transcriptional response in mesangial cells	[43,45]
SUMO1/2/3	positive	Ras	ERK activation	[54]
		PML	promotes formation of PML-NBs	[111,112]
			enhances P-Smad2/3 and TGF-β1 mRNA expression	
		Elk-1	nuclear export	[60,63]
			recruitment of histone deacetylase activity to promoters	
SUMO1/2	negative	PI3K p85	phosphorylation of PI3K	[76]
	positive	PI3K p110	activation of AKT	[77]
			stability	

## 14. Discussion and outlook

Many studies show increased SUMOylation in fibrotic organs, which suggests that SUMOylation serves as an inducer of fibrogenesis [31,141,142]. It is a well-accepted view that TGF-β signalling plays an important role in the process of fibrosis, and many key molecules are substrates for SUMOylation. It is not surprising that the effect of SUMOylation on various proteins differs. Although many studies show that SUMO modification promotes TGF-β-induced fibrosis [143], some proteins, such as Smad4, MEK, TAB2, PTEN, PI3K, c-Jun and SNIP, exhibit inhibitory effects on TGF-β signalling after being SUMOylated.

We attempted herein to explain observations using the MAPK family as an example. The MAPK signalling pathway involves ERK, JNK and p38 protein families. Although p38 is not considered a member of the non-canonical pathway of the TGF-β pathway, recent studies indicate that the p38 pathway is stimulated by TGF-β1 to induce fibrosis. p38 has also been demonstrated to inhibit nuclear translocation through non-covalent SUMO–p38 interactions [144]. Therefore, we propose that, although SUMO modification has a variety of cytological functions, the change in protein function after SUMOylation may be associated with the position of the protein in the signalling pathway. In the MAPK family, SUMO modification is biased towards inhibiting signalling pathway activation by recruiting transcription inhibitors to downstream targets of ERK, JNK and p38 [63]. SUMOylation of the upstream protein may be biased to inhibit its phosphorylation, which destroys its ability to activate downstream proteins [93]. The role of proteins located in the middle of the signalling pathway does not seem to be altered by SUMOylation, but the abilities of these proteins to regulate other proteins vary. In addition, the wide and complex cross-talk occurring between signalling pathways is another explanation; as mentioned above, ERK is involved in the activity of Smad3 [67].

The pathogenesis of fibrosis is not restricted to fibroblast differentiation and ECM deposition. Autophagy is an evolutionarily conserved cellular catabolic pathway responsible for delivering long-lived proteins and excess or damaged organelles to the lysosome for degradation and re-use of the resulting macromolecules [145]. This characteristic makes autophagy a key player in cellular homeostasis, and this process is expected to become a new target for the treatment of fibrosis [146,147]. Although the mechanism of autophagy remains unclear, the role of PI3K/Akt/mTOR signalling as the primary autophagy regulatory pathway is widely accepted [145]. mTOR inhibits autophagy by decreasing phosphorylation levels of the autophagy-related protein Unc-51-like kinase [148]. Class III phosphatidylinositol 3-kinase (PtdIns3 K) activity can be opposed by PTEN, with subsequent mTOR inhibition [145]. JNK has also been shown to promote the induction of autophagy [149]. In view of the inhibitory effect of SUMOylation on PI3K and the promotional effect of SUMOylation on JNK, autophagy may constitute another target by which SUMOylation regulates fibrosis.

## 15. Conclusion

In this review, we summarize the role of SUMOylation in Smad and non-Smad TGF-β signalling pathways and propose that SUMOylation is important in TGF-β-mediated biological processes. Most studies to date on the effect of SUMO modification on TGF-β pathway activity have focused on cancer. Considering that EMT is indispensable for fibrogenesis and tumorigenesis and biomarkers such as MMP and α-SMA are commonly employed to determine whether a drug is effective as a fibrosis or cancer treatment, we suggest that SUMOylation may be used in a novel fibrosis treatment through the inhibition of EMT. It is worth mentioning that fibrosis is important in the premalignant environment, especially in liver fibrosis; that is, cancer can be considered a negative outcome of fibrosis to some extent [150]. In view of the common characteristics of the pathological mechanisms of cancer and fibrosis, anti-tumour drugs that inhibit SUMOylation of TGF-β pathway components may be used when certain organs begin to undergo fibrosis [151]. Ultimately, the goal of reversing fibrosis and preventing cancer may be achieved, which indicates that SUMO inhibitors may achieve two goals at the same time.

## Data accessibility

This article has no additional data.
